# Associations of Serum Nitric Oxide with Vitamin D and Other Metabolic Factors in Apparently Healthy Adolescents

**DOI:** 10.1155/2018/1489132

**Published:** 2018-08-05

**Authors:** Nasser M. Al-Daghri, Ihtisham Bukhari, Sobhy M. Yakout, Shaun Sabico, Malak N. K. Khattak, Ibrahim Aziz, Majed S. Alokail

**Affiliations:** Prince Mutaib Chair for Biomarkers of Osteoporosis and Biomarkers Research Program, Biochemistry Department, College of Science, King Saud University, Riyadh 11451, Saudi Arabia

## Abstract

**Introduction:**

Nitric oxide (NOx) is an important biomolecule which interacts with other molecules including 25(OH)D to mediate various metabolic pathways. Interactions and associations of NOx with 25(OH)D have been well studied both* in vitro* and* in vivo*, yet associations in apparently healthy adolescents have never been studied.

**Methods:**

A total of 740 (245 boys and 495 girls) apparently healthy Saudi adolescents aged 10-17 years were included in this cross-sectional study, to determine the associations of NOx with 25(OH)D and other biomarkers in Saudi adolescents. Serum NOx, 25(OH)D, and other biochemical and anthropometric parameters were measured following standard protocols and manufacturers' guidelines.

**Results:**

NOx level was significantly higher in boys than girls (*p*<0.001). In all subjects, NOx showed a significant inverse correlation with 25(OH)D. After stratification according to sex however this significant association was observed only in boys and not in girls. NOx was also significantly associated with BMI, serum triglycerides, and systolic blood pressure in all subjects.

**Conclusion:**

The significantly inverse association of NOx and 25(OH)D among apparently healthy adolescents is influenced by sex and further strengthens the extraskeletal role of 25(OH)D in maintaining endothelial homeostasis in this age group, particularly in boys. Whether vitamin D correction can influence NOx production over time among adolescents remains to be proven.

## 1. Introduction

Nitric oxide (NOx) is an important molecule produced by endothelium which plays a variety of vital roles in humans such as reproduction, inflammation, vasodilation, cardiac function, oxidative stress, gene transcription, translation, and posttranslational modifications of the proteins [1–6]. Exercise and mental stress reduce bioactivity of NOx which leads to constriction of coronary arteries. Reduced NOx level also facilitates vascular inflammation which may cause oxidation of lipoproteins and foam cell formation, the precursor of atherosclerotic plaque [7]. The disturbance of its endothelial function may lead to hypercholesterolemia, systemic hypertension, diabetes, congestive heart failure, atherosclerosis, pulmonary hypertension, estrogen deficiency, hyperhomocysteinemia, and aging [8]. NOx is synthesized from L-arginine, nitric oxide synthase's (NOS) activity which requires calcium/calmodulin [9, 10]. Calcium-calmodulin complex is formed by the binding of calcium to calmodulin that further binds with the latch domain. The transfer of electron from NADPH via flavin is facilitated by this group which further converts L-arginine and O2 to L-citrulline and NOx [11, 12]. Vitamin D responds to NOS gene which suggests that both molecules have functional association [13]. The active vitamin D (1,25-dihydroxyvitamin D [1,25(OH)2D]) [14] regulates the production of NOx and/or expression of inducible NOS (iNOS) in different cells including endothelial cells, osteoblasts, microglial cells, macrophages, and astrocytes [13, 15]. Associations of NOx with vitamin D and its pathogenicity have been well studied in cell lines and animal models [13, 15, 16]. It has also been measured in some chronic diseases but mostly in adults. To the best of our knowledge, no similar report is found in apparently healthy adolescents. Given the importance of NOx and the extra-skeletal effects of vitamin D in the biological system, the present study was conducted to investigate the associations of NOx with 25(OH)D and various cardiometabolic factors in apparently healthy adolescents of Saudi Arabia.

## 2. Materials and Methods

### 2.1. Subjects

A total of 740 (245 boys and 495 girls) Saudi adolescents (10-17 years) were included in this cross-sectional study. Subjects' information regarding vitamin D was taken from the school project master database of the Prince Mutaib Chair for the Biomarkers of Osteoporosis (PMCO) in King Saud University (KSU) [17, 18]. In brief, all subjects were recruited from the different primary schools in Riyadh, Saudi Arabia. Physical examination of the individuals was done to ensure the inclusion criteria; the individuals with serious clinical conditions like chronic kidney or liver diseases, cardiovascular diseases, neurological problems, bone problem, and drugs abusers were excluded from the study. Individuals taking/taken vitamin D supplementation and those taking treatment for other severe illness were also excluded from study. Anthropometric data was also recorded including height (rounded off to the nearest 0.5cm), weight (rounded off to the nearest 0.1kg), waist and hip circumference (centimeters), and mean systolic and diastolic blood pressure (millimeters of Hg). Body mass index (BMI) was calculated as weight (kg) divided by height in square meters and individuals with BMI ≥25kg/m^2^ and ≥30kg/m^2^ were considered overweight and obese, respectively. We excluded subjects who were underweight, overweight, and obese, based on their BMI z-scores. We recruited only apparently normal individuals for current study.

### 2.2. Sample Collection

A total 5-10 CC fasting venous blood samples were collected from all subjects. Part of the blood samples were centrifuged for serum isolation while remaining blood samples were transferred to EDTA tubes for other future analyses. Blood and serum samples were immediately delivered and stored at −80°C in PMCO, KSU, Riyadh, KSA.

### 2.3. Biochemical Analyses

Fasting lipid profile and blood glucose in all recruited individuals were determined using a chemical analyzer (Konelab 20XTi, Thermo Electron Corporation, Vantaa, Finland). COBAS e-411 automated analyzer (Roche Diagnostics, Indianapolis, IN, USA) was used for measuring serum 25(OH)D. The inter- and intra-assay were applied for estimation of serum 25(OH)D; coefficients of variation (CV) were taken as 8.0 and 5.6%, respectively, with a lower detection limit (LOD) of 7.5nmol/ml [19].

Usually, NOx can be estimated from determining the concentrations of nitrite and nitrate end products. The measurement of nitrate/nitrite concentration or of total nitrate and nitrite concentration is routinely used as an index of NOx production [15]. The concentration of serum NOx was measured using the Griess reaction [20]. Because of its simplicity, it has been used extensively in analysis of numerous biological samples including plasma (serum), urine, CSF, saliva, and cell culture media. In this method, nitrite is first treated with a diazotizing reagent, e.g., sulfanilamide (SA), in acidic media to form a transient diazonium salt. This intermediate is then allowed to react with a coupling reagent, N-naphthyl-ethylenediamine (NED), to form a stable azo compound that can be measured spectrophotometry. In brief, 50 *μ*L serum samples and 50 *μ*L of distilled water (blank) were added to microplate wells, and 50 *μ*L sulfanilamide (1% in 5% H3PO4) was added to samples. Samples were incubated for 10 min at 37°C. 50 *μ*L of [N-(1-naphthyl) ethylendiamine dihydrochloride (0.1%) in distilled water] was then added to both samples and blank and reincubated for 5 min at 37°C. Absorbance was taken at 540 nm using the enzyme-linked immunosorbent assay (ELISA) reader (Sunrise, Tecan, Austria). Serum NOx concentration was determined from the linear standard curve established using 0-50*μ*M sodium nitrate. Inter- and intra-assay coefficients of variations were set at 5.2% and 4.4%, respectively, while the recovery of the assay was 93±1.5%.

### 2.4. Statistical Analysis

SPSS (version 21.0, IBM) was used to analyze the data. Continuous data were presented as mean ± standard deviation (SD) for normal variables and non-Gaussian variables were presented in median (1st and 3rd) percentiles. Categorical data were presented as frequencies and percentages (%). All continuous variables were checked for normality using Kolmogorov-Smirnov test. Non-Gaussian variables were log-transformed prior to parametric analysis. Correlations between variables were done using Pearson's correlation analysis. Independent T-test was used to compare mean differences. A* p* value <0.05 was considered statistically significant.

## 3. Results 

**Figure 1:**
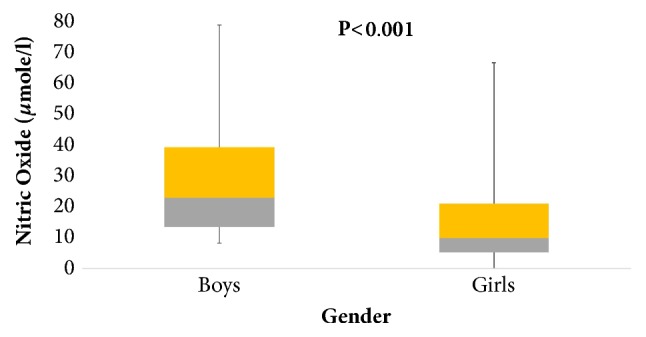
Data is presented in median (1^st^-3^rd^) percentiles. *∗∗∗* represents overall significance at 0.01 level.

**Figure 2:**
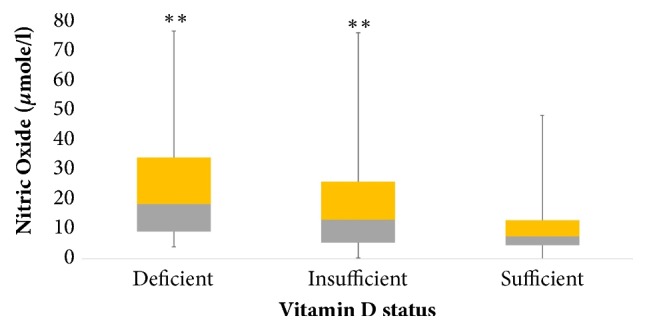
Data is presented in median (1^st^-3^rd^) percentiles. *∗∗∗* represents overall significance at 0.01 level; *∗∗* represents significance between deficient and insufficient ones at 0.01 level.

A total of 740 (245 boys and 495 girls) were recruited to analyze the biological relation of NOx with vitamin D and other serological parameters. Initially, the general characteristics of subjects were studied and no significant differences were found between the mean age and triglycerides. Significantly higher BMI and hip circumference were noted in boys (*p*<0.001) while girls had significantly higher WHR and waist circumference (*p*<0.001). Girls also had significantly higher systolic blood pressure while boys had significantly higher diastolic blood pressure. Girls showed significantly higher levels of serum total cholesterol and 25(OH)D while the boys had significantly higher levels of triglycerides and NOx (*p*<0.001) (Table 1). The sexually dimorphic associations between vitamin D and NOx levels are shown in Figure 1. Furthermore, the bivariate associations of NOx were studied with other parameters like age, blood pressure, total cholesterol, and triglycerides. The mean age, systolic blood pressure, and triglycerides had positive correlation while total cholesterol and HDL-cholesterol showed inverse correlation with NOx. The NOx in girls showed a significant positive correlation between hip circumference, systolic blood pressure, and triglycerides while inverse correlations were found between cholesterol, HDL, and serum 25(OH)D. In all subjects, NOx did not show an association with BMI, waist, WHR, and diastolic blood pressure and glucose (Table 2). The differences of vitamin D status in subjects were measured according to the values analyzed previously [21] as sufficient, deficient, and insufficient (75nmol/L, >50 and <75nmol/L, and <50nmol/L), respectively. There were no significant differences between mean glucose, systolic blood pressure, HDL/ LDL, LDL/HDL ratio, and triglycerides in all groups. Significantly higher BMI, hip circumference, diastolic blood pressure, and NOx were observed in vitamin D deficient group (*p* value<0.001), whereas the vitamin D sufficient group had significantly higher mean values of WHR, total cholesterol, and vitamin D (Table 3). Vitamin D insufficient group showed a significant positive correlation with hip circumference, systolic blood pressure, and triglycerides. Triglycerides also showed a positive association with deficient and sufficient group while LDL/HDL ratio was found only in the deficient group. Inverse associations with total and HDL- and LDL-cholesterol were found and WHR was inversely associated with NOx in the insufficient group (Table 4). For confirmation, based on the vitamin D status (75nmol/L, >50 and <75nmol/L, and <50nmol/L) all individuals were grouped into three categories (sufficient, deficient, and insufficient), respectively. It was found that individuals having low vitamin D levels (insufficient group) had higher levels of NOx (Figure 2).

## 4. Discussion

Vitamin D and NOx are important molecules that may have a functional association as observed in different cell lines, animal models, and humans. Different studies showed different associations, so this ambiguity has been a hot topic that remains unanswered. NOx has a role in the pathogenicity of several illnesses like neurodegenerative diseases (e.g., excitotoxicity following stroke, multiple sclerosis, Alzheimer's, and Parkinson's diseases asthma), CVD, arthritis, and infertility [6, 22–25]. NOx also has a protective role against osteoarthritis [26].

It has been observed that 1,25(OH)2D3 reduces PFF-induced NOx response in primary human osteoblasts while 25(OH)D3 did not show significant effects on NOx response in primary human osteoblasts [16]. Without the action of mechanical stimuli like vitamin D receptor, 1,25(OH)(2)D(3) improves the production of NOx and expression of inducible-NOS osteoblasts [13]. The addition of 25(OH)D to HD11 cells and monocytes in the presence of lipopolysaccharide (LPS) increases nitrate production 3-5-fold approximately. Consistent increase in nitrate production was reported in HD11 cells with the consistent increase in doses of 25(OH)D [27]. It has also been noted that addition of vitamin D increases production of endothelial NOx [15]. By adding vitamin D to the diet of atherosclerotic rabbits, a significant increase was noted in NOx production. Hence, authors concluded that vitamin D has antiatherosclerotic effects and stimulate NOx production which attenuates the inflammatory atherosclerotic process [28]. A significant direct relationship was noted between NOx and vitamin D in African-American men [29]. On the other hand, contradictory results were shown in various studies; the functional relation of vitamin D and exhaled NOx in lungs was studied in children (ages= 5-18) but no significant association was found. Similarly, no association was shown between 25(OH)D and exhaled NOx in children with asthma [30]. Vitamin D reduces asthma by restricting NOx production and expression of iNOS in lungs [31]. Similar results were found in cell line RAW 264.7 in which increasing doses of 1,25(OH)(2)D(3) inhibited iNOS messenger RNA expression and the production of NOx was also reduced with increasing vitamin D. Even no association was found between vitamin D binding protein (DBP) and NOx production by mononuclear cells [32]. The negative correlation was found between the NOx production and level of 1,25(OH)(2)D(3) in peripheral-blood mononuclear cells (PBMCs) taken from hypercalcemia and tuberculosis patients [33]. Similarly, current findings showed an inverse association between vitamin D and NOx. While NOx has shown positive associations between high systolic blood pressure and triglycerides in the girls group, no associations were found with total cholesterol, HDL, and vitamin D. Inverse association has been shown in NOx and systolic blood pressure among patients with myocardial infraction [34]. In pregnant women with hypertension, NOx synthesis was significantly reduced with increased levels of triglycerides and cholesterol [35]. Current individuals having high/low level of NOx and vitamin D were apparently normal adolescents, without any sign of bone or other serious diseases previously associated with NOx or vitamin D production. The associations between NOx, high systolic blood pressure, and triglycerides have not been studied previously in healthy adolescents.

Although our findings are similar to previous studies and the relatively large sample size had sufficient statistical power to explain the relationship between NOx and other parameters, the study still has some limitations. One is its cross-sectional design, which precludes our ability to make causal inferences about the observed associations. We also did not measure levels of lactate and other inflammatory markers which may give additional insights into the significant associations elicited. These markers can be recommended for further investigations. Furthermore since our subjects were adolescents, we did not study the involvement of growth hormones and sex steroids, which are suggested to have influence on NOx and 25(OH)D levels. Finally, since the associations observed do not prove causality, a sufficient vitamin D status may, at best, serve as a “marker” for other conditions, e.g., for good health or for UV exposure. In consequence, it can be speculated whether the associations observed may be caused by other, unknown factors that are associated, e.g., with good health or UV exposure.

In summary, the associations of NOx with 25(OH)D and other cardiometabolic parameters were studied in 740 Saudi adolescents. Serum 25(OH)D and NOx showed a significant inverse association in all subjects, more so in boys, and NOx was positively associated with triglycerides and systolic blood pressure. The association between the NOx and the parameters measured are significantly influenced by sex and age. A large scale study on people of different ages and ethnicity would help in better understanding of the NOx's association pattern with metabolic and clinical factors.

## Figures and Tables

**Table 1:** Clinical characteristics of the subjects.

**Parameter**	**All**	**Boys**	**Girls**	**P-Value**
**N**	740	245 (33.1)	495 (66.9)	
Age (years)	14.2 ± 1.6	14.1 ± 1.2	14.3 ± 1.8	0.18

Body Mass Index (kg/m^2^)	21.9 ± 4.8	22.7 ± 4.4	21.5 ± 4.9	**0.003**

Waist circumference (cm)	73.6 ± 11.7	72.2 ± 10.0	74.7 ± 12.8	**0.011**

Hip circumference (cm)	88.0 ± 11.2	92.8 ± 10.1	84.3 ± 10.6	**<0.001**

WHR	0.8 ± 0.09	0.8 ± 0.1	0.9 ± 0.1	**<0.001**

Systolic BP (mmHg)	119.4 ± 14.0	116.0 ± 13.0	121.0 ± 14.2	**<0.001**

Diastolic BP (mmHg)	70.1 ± 9.8	72.6 ± 10.2	68.9 ± 9.3	**<0.001**

Glucose (mmol/l)	5.1 ± 0.7	4.9 ± 0.8	5.2 ± 0.7	**<0.001**

Triglycerides (mmol/l)#	1.0 (0.8-1.4)	1.0 (0.8-1.4)	1.0 (0.8-1.4)	0.62

Total Cholesterol (mmol/l)	4.3 ± 1.0	4.0 ± 1.1	4.4 ± 0.9	**<0.001**

HDL-Cholesterol (mmol/l)	1.2 ± 0.4	1.0 ± 0.2	1.3 ± 0.4	**<0.001**

LDL-Cholesterol (mmol/l)	2.6 ± 0.8	2.6 ± 0.8	2.6 ± 0.8	**0.310**

LDL/HDL ratio	2.5 ± 1.2	2.7 ± 0.9	2.4 ± 1.3	**0.004**

25(OH)D (nmol/l)#	33.6 (24.9-46.6)	25.6 (20.2-32.2)	38.6 (29.7-50.2)	**<0.001**

Nitric Oxide (*µ*mol/l)#	12.8 (5.7-26.4)	21.9 (12.8-37.5)	9.3 (5.0-20.0)	**<0.001**

**Note:** data is presented as N (%) for frequencies and mean ± standard deviation for normal continuous variables; # denotes non-Gaussian variables presented as median (25^th^-75^th^) percentiles; P-value is significant at 0.05.

**Table 2:** Association between NOx and other parameters.

**Parameters**	**All**	**Boys**	**Girls**
N (%)	740	245 (33.1)	495 (66.9)

Age (years)	0.01	0.25∗∗	0.06

Body Mass Index (kg/m^2^)	0.10∗	0.07	0.04

Waist circumference (cm)	0.09∗	0.03	0.10

Hip circumference (cm)	0.20∗∗	0.03	0.12∗

WHR	-0.12∗∗	-0.04	0.04

Systolic Blood Pressure (mmHg)	0.11∗∗	0.33∗∗	0.13∗∗

Diastolic Blood Pressure (mmHg)	0.10∗	-0.04	0.08

Glucose (mmol/l)	-0.06	-0.03	0.03

Triglycerides (mmol/l)#	0.26∗∗	0.25∗∗	0.31∗∗

Total Cholesterol (mmol/l)	-0.36∗∗	-0.38∗∗	-0.29∗∗

LDL-Cholesterol (mmol/l)	-0.22∗∗	-0.11	-0.26∗∗

LDL/HDL ratio	0.07	0.15∗	-0.01

HDL-Cholesterol (mmol/l)	-0.38∗∗	-0.38∗∗	-0.27∗∗

25(OH)D (nmol/l)#	-0.21∗∗	0.04	-0.37∗∗

**Note**: data is presented as coefficient (R); # denotes log transform; ∗ denotes significance at 0.05 level; ∗∗ denotes significance at 0.01 level.

**Table 3:** Clinical characteristics of the subject.

**Parameter**	**Deficient**	**Insufficient**	**Sufficient**	**P-Value**
N (%)	186 (25.1)	418 (56.5)	136 (18.4)	
Age (years)	14.5 ± 1.5	14.2 ± 1.5	13.4 ± 1.8^*∗*!^	<0.001

Body Mass Index (kg/m^2^)	22.7 ± 4.8	22.0 ± 4.7	19.9 ± 3.9^*∗*!^	<0.001

Waist circumference (cm)	73.0 ± 10.8	74.3 ± 11.7	70.0 ± 12.1^!^	0.010

Hip circumference (cm)	91.0 ± 10.8	87.5 ± 9.7^*∗*^	80.5 ± 11.3^*∗*!^	<0.001

WHR	0.8 ± 0.1	0.8 ± 0.1	0.9 ± 0.1	<0.001

Systolic BP (mmHg)	119.1 ± 14.5	119.9 ± 13.2	117.2 ± 15.4	0.23

Diastolic BP (mmHg)	72.2 ± 10.1	69.9 ± 9.4^*∗*^	68.2 ± 9.6^*∗*!^	0.005

Glucose (mmol/l)	5.1 ± 0.8	5.2 ± 0.7	5.0 ± 0.6	0.12

Triglycerides (mmol/l)#	1.1 (0.8-1.5)	1.0 (0.8-1.3)	1.0 (0.8-1.3)	0.13

Total Cholesterol (mmol/l)	4.1 ± 1.1	4.3 ± 1.0	4.5 ± 1.0^*∗*^	0.015

HDL-Cholesterol (mmol/l)	1.2 ± 0.4	1.2 ± 0.4	1.2 ± 0.4	0.42

LDL-Cholesterol (mmol/l)	2.5 ± 0.7	2.6 ± 0.8	2.7 ± 0.9	0.22

LDL/HDL ratio	2.4 ± 0.9	2.4 ± 1.2	2.6 ± 1.5	0.17

25(OH)D (nmol/l)#	20.4 (18.3-22.5)	35.1 (30.0-49.5)^*∗*^	59.8 (53.2-65.5)^*∗*!^	<0.001

Nitric Oxide (*µ*mole/l)#	18.0 (8.9-33.5)	12.9 (5.2-25.4)^*∗*^	7.4 (4.4-12.7)^*∗*!^	<0.001

**Note:** data is presented as N (%) for frequencies and mean ± standard deviation for normal continuous variables; # denotes continuous variables with non-Gaussian distribution presented as median (25^th^-75^th^) percentiles; P-value is significant at 0.05 and 0.01. “**∗**” denotes significance compared to deficient; “**!**” denotes significance compared to insufficient.

**Table 4:** Association between nitric oxide with other parameters according to vitamin D status.

**Parameter**	**Deficient**	**Insufficient**	**Sufficient**
N	186 (25.1)	418 (56.5)	136 (18.4)

Age (years)	0.03	-0.003	0.08

Body Mass Index (kg/m^2^)	-0.01	0.06	0.04

Waist circumference (cm)	0.05	-0.04	0.10

Hip circumference (cm)	0.05	0.17∗∗	0.05

WHR	-0.003	-0.26∗∗	0.14

Systolic BP (mmHg)	0.11	0.11∗∗	0.08

Diastolic BP (mmHg)	0.13	0.10	-0.12

Glucose (mmol/l)	-0.03	-0.08	-0.05

Triglycerides (mmol/l)#	0.20∗	0.32∗∗	0.24∗

Total Cholesterol (mmol/l)	-0.36∗∗	-0.34∗∗	-0.26∗∗

HDL-Cholesterol (mmol/l)	-0.39∗∗	-0.41∗∗	-0.22∗∗

LDL-Cholesterol (mmol/l)	-0.19∗	-0.18∗∗	-0.29∗∗

LDL/HDL ratio	0.21∗	0.11	-0.09

25(OH)D (nmol/l)#	0.06	-0.04	-0.04

**Note**: data is presented as coefficient (R); # denotes log transform; ∗ denotes significance at 0.05 level; ∗∗ denotes significance at 0.01 level.

## Data Availability

The data used to support the findings of this study are available from the corresponding author upon request.

## Abbreviations

NO: Nitric oxide
25(OH)D: 25-Hydroxyvitamin D
CVD: Cardiovascular diseases
HDL: High quality lipoproteins
LDL: Low quality lipoproteins
WHR: Waist hip ratio
BMI: Body mass index.
